# Changes in the Bristle Stiffness of Polybutylene Terephthalate Manual Toothbrushes over 3 Months: A Randomized Controlled Trial

**DOI:** 10.3390/ma13122802

**Published:** 2020-06-22

**Authors:** Yoshino Kaneyasu, Hideo Shigeishi, Kouji Ohta, Masaru Sugiyama

**Affiliations:** Department of Public Oral Health, Program of Oral Health Sciences, Graduate School of Biomedical and Health Sciences, Hiroshima University, Hiroshima 734-8553, Japan; d186958@hiroshima-u.ac.jp (Y.K.); otkouji@hiroshima-u.ac.jp (K.O.); masaru@hiroshima-u.ac.jp (M.S.)

**Keywords:** toothbrush, polybutylene terephthalate, bristle stiffness, bristle splaying, randomized controlled trial

## Abstract

We previously reported that polybutylene terephthalate (PBT) toothbrushes become less effective for plaque removal after two months of use. However, it remains unknown how the bristle stiffness of PBT toothbrushes changes after several months of use. We performed a single-center randomized controlled trial to evaluate the bristle stiffness and bristle splaying of soft and medium manual toothbrushes among dental and medical students of Hiroshima University. Subjects were 80 participants who met the criteria. Participants were randomly assigned to the soft toothbrush group (n = 40) or the medium toothbrush group (n = 40). We collected toothbrushes immediately after first use (T0), after one month of use (T1), after two months of use (T2), and after three months of use (T3). Bristle stiffness was measured according to the International Organization for Standardization (ISO) 22254. The mean bristle stiffness (N/cm^2^) of soft and medium toothbrushes was significantly lower at T2 and T3 than at T0 (T2 vs. T0, soft; 3.63 vs. 3.89, P < 0.01 and medium; 4.33 vs. 4.52, P < 0.05, respectively, and T3 vs. T0, 3.62 vs. 3.89, *p* < 0.01 and 4.18 vs. 4.52, *p* < 0.001, respectively). Bristle stiffness was significantly reduced in soft and medium PBT toothbrushes after two months of use.

## 1. Introduction

Tooth brushing plays an essential role in the removal of dental plaque and the prevention of gingival inflammation [1]. However, toothbrushes begin to wear out and become less effective in cleaning teeth over time [2,3]. We previously reported that polybutylene terephthalate (PBT) toothbrushes become less effective in removing dental plaque after two months of use [4]. Therefore, regular toothbrush replacement is recommended to maintain good oral hygiene. A proper brushing technique is required to remove dental plaque without damaging the tooth surface and gingiva. The bristle stiffness of a toothbrush may also be involved in the efficacy and safety of plaque removal. 

There are several methods for measuring the bristle stiffness of a toothbrush [5]. Rawls et al. reported a mathematical method to predict bristle stiffness [6], and Yankell et al. reported a method for measuring bristle stiffness in diamond-shaped toothbrush filaments according to the International Organization for Standardization (ISO) [7]. ISO 22254 was confirmed in 2015 and is applicable to measuring the resistance to deflection of the tufted portion of a toothbrush (i.e., conventional manual toothbrushes with a flat trim design) [8]. The resistance of the tufted portion to deflection is used to assess the bristle stiffness of a toothbrush. Therefore, we employed the ISO 22254 test for the assessment of bristle stiffness in this study.

Several laboratory studies have investigated the relationship between bristle stiffness and tooth abrasion [9,10,11,12]. However, it remains unknown how the bristle stiffness of a PBT toothbrush changes after a few months of use. Additionally, the association of bristle stiffness with bristle splaying has not been fully investigated in PBT toothbrushes. Therefore, we aimed to investigate the changes in the bristle stiffness of PBT toothbrushes after one, two, and three months of use. The relationships between bristle stiffness and the degree of bristle splaying were also investigated.

## 2. Materials and Methods 

### 2.1. Study Design 

We performed a single-center randomized controlled trial (RCT) to evaluate the bristle stiffness and bristle splaying of soft and medium manual toothbrushes among dental and medical students of Hiroshima University [4]. The study was performed at the Department of Public Oral Health, Graduate School of Biomedical and Health Sciences, Hiroshima University. The Ethical Committee of Hiroshima University approved the study design (No. C-120), and all participants signed informed consent agreements. We registered this clinical trial in the UMIN Clinical Trials Registry System (UMIN000025133). The participants were recruited from November 2016 to September 2017. We included a total of 80 participants who met the criteria for this RCT. The sample size required for the t-test was calculated with a statistical power of 80% and a significance level of 5%, with a maximum loss to follow-up of 10%; we found that a group size of 37 subjects was required. Therefore, we aimed to analyze 40 participants per group. Eligibility criteria and participant characteristics have also been described in our previous paper [4]. Eligible participants were Hiroshima University students who were ≥18 years of age and <40 years of age, with at least 18 natural teeth excluding the third molars. Exclusion criteria were as follows: subjects who needed dental treatment, were receiving prosthodontic treatment, or were pregnant. Participants were then randomly assigned to the soft toothbrush group (n = 40) or the medium toothbrush group (n = 40) with a 1:1 allocation. The method of random allocation was described in our previous paper [4]. Before the start of this study, a dental hygienist provided the participants with instructions for toothbrushing. Participants were instructed to brush their teeth using a gentle scrubbing technique [4]. Participants were also instructed to brush their teeth for 3 minutes twice per day with an allocated toothbrush. Eight participants discontinued the intervention for personal reasons unrelated to the program (n = 6 in the medium toothbrush group and n = 2 in the soft toothbrush group).

Two manual toothbrushes with different bristle stiffness (soft bristles and medium bristles) were used in this study. Participants in the soft toothbrush group used a soft toothbrush (Tuft24, soft, Oral Care Ltd., Tokyo) and those in the medium toothbrush group used a medium toothbrush (Tuft24, medium, Oral Care Ltd., Tokyo). We collected toothbrushes immediately after first use (T0: baseline), after 1 month of use (T1: month 1), after 2 months of use (T2: month 3), and after 3 months of use (T3: month 6), following the allocation of new toothbrushes. 

### 2.2. Manual Toothbrushes 

The toothbrushes had three rows of bristles with a total of 24 tufts and a height of 9.0 mm. The Tuft24 (soft) bristles contained approximately 37 monofilaments per tuft, and the diameter of the monofilaments was 0.15 mm. The Tuft24 (medium) bristles contained approximately 20 monofilaments per tuft and the diameter of the monofilaments was 0.2 mm.

### 2.3. Measurement of the Resistance of the Tufted Portion to Deflection

The resistance to deflection of the tufted portion was measured by ISO 22254. A toothbrush stiffness testing machine (Japan MECC Co. Ltd., Tokyo, Japan) was employed to measure the bending resistance of the tufted portion of the bristle. The testing machine conformed to the ISO 22254 standard and was composed of a brushing head, brushing table, gripping block, and load cell. The brushing table had five stainless steel bars with a diameter of 3.2 ± 0.1 mm. 

Before the bending resistance of the tufted portion was measured. The weight of the toothbrush head was adjusted to be approximately 5.0 g. Next, the toothbrush head was soaked in 37 ± 2 °C water for 90 seconds. The toothbrush head was shaken to remove excess water, and then fixed to the gripping block. We confirmed that the surface of the toothbrush head was parallel to the brushing table on the machine. This machine provided reciprocating movements of the toothbrush head at a speed of 10 mm per second. The toothbrush head was loaded with a force of 5 ± 0.05 Newtons (N) to make the bristles slip on the brushing table. The reaction force caused by the deflection of the tufted portion was recorded when the bristles moved back and forth five times. The average of the maximum value in the two directions (i.e., the back and forth directions) was calculated as the resistance to deflection force of the bristles. In accordance with previous study, the resistance to deflection force of the bristles (N) and the tufted area of the toothbrush (i.e., the area surrounded by a peripheral tangent line to the outer tufts) (cm²) was used to evaluate the toothbrush stiffness [8]. The toothbrush stiffness was obtained with the following equation: Toothbrush stiffness (N/cm²) = resistance to the deflection force of the bristles (N)/the tufted area (cm²) [8]. 

### 2.4. Measurement of the Wear Index Using Digital Vernier Calipers

The degree of toothbrush splaying was evaluated with the wear index developed by Rawls et al. and improved by Sforza et al. [13,14]. The width of the toothbrush (i.e., the brush width at the brushing surface, as viewed from the lateral side (W1), the brush width at the base of the bristles, as viewed from the lateral side (W2), the brush width at the brushing surface, as viewed from the frontal side (W3), the brush width at the base of bristles, as viewed from the frontal side (W4), the maximum brush trim height before toothbrush use, or the length of the highest standing bristle after toothbrush use (L)) was measured using a digital vernier caliper (Shinwa Measuring Tools Corp., Niigata, Japan). The wear index was calculated using the following equation: Wear index = (W1 − W2 + W3 − W4)/L. The size of the toothbrush was measured by one examiner. One examiner, who did not know how long the toothbrush had been used, assessed the wear index using a randomly selected toothbrush. The intrarater reliability of the examiner was assessed using an intraclass correlation coefficient. An examiner calculated the wear index of five toothbrushes twice each. The calculated value of the intraclass correlation coefficient was 0.953, suggesting that the examiner had excellent reliability according to the reported criteria for intraclass correlation coefficients [15].

### 2.5. Statistical Analysis

The results were analyzed statistically using SPSS software (version 25; IBM Corp., Japan, Inc.). Tukey’s honestly significant difference (HSD) test or the Mann–Whitney U test with Bonferroni correction was used for multiple comparisons to compare toothbrush stiffness or wear index changes. Correlations between toothbrush stiffness and the wear index were analyzed using Spearman’s rank correlation coefficient. *p* < 0.05 was considered to indicate statistical significance.

## 3. Results

### 3.1. Changes in the Bristle Stiffness of Soft and Medium Toothbrushes

The bristle stiffness of toothbrushes was measured at T0, T1, T2, and T3. Medium toothbrushes exhibited higher bristle stiffness than soft toothbrushes at T0, T1, T2, and T3 (Table 1). The stiffness of soft toothbrushes was significantly decreased at T2 (3.63 ± 0.29) and T3 (3.62 ± 0.32) when compared with T0 (3.89 ± 0.29) (Table 1). The bristle stiffness of medium toothbrushes was significantly decreased at T2 (4.33 ± 0.26) and T3 (4.18 ± 0.25) when compared with T0 (4.52 ± 0.30) (Table 1).

### 3.2. Changes in the Wear Index of Soft and Medium Toothbrushes 

We examined the wear index of soft and medium toothbrushes at T0, T1, T2, and T3. Soft toothbrushes exhibited a higher mean wear index than medium toothbrushes at T0, T1, T2, and T3 (Table 2). The mean wear index of soft toothbrushes was significantly higher at T1, T2, and T3 than at T0 (Table 2). The mean wear index of medium toothbrushes was significantly higher at T1, T2, and T3 than at T0 (Table 2). 

### 3.3. Correlation between Bristle Stiffness and the Wear Index 

A correlation between bristle stiffness and the wear index was investigated in soft and medium toothbrushes. There was a significant negative weak correlation between bristle stiffness and the wear index in soft toothbrushes (ρ = −0.362, *p* < 0.001). Additionally, a significant negative weak correlation was found between bristle stiffness and the wear index in medium toothbrushes (ρ = −0.374, *p* < 0.001).

## 4. Discussion

Toothbrush bristle stiffness is dependent on the stiffness of the component bristles (i.e., natural bristles made of bamboo, hog or boar hair; and synthetic bristles made of nylon or PBT) [16]. Additionally, several factors such as bristle diameter, bristle length, the number of tufts, and the number of bristles per tuft are importantly associated with bristle stiffness [13,17]. Nylon toothbrushes with large diameter bristles had higher stiffness than those with small diameter bristles [6], indicating that the diameter of the bristle is importantly related to the bristle stiffness of nylon toothbrushes. Previously, the bristle stiffness of toothbrushes has been categorized according to the diameter of single bristles (i.e., 0.2 mm, soft; 0.3 mm, medium; 0.4 mm, hard) [17]. In this study, medium PBT toothbrushes with 0.2 mm diameter bristles exhibited higher stiffness than soft toothbrushes with 0.15 mm diameter bristles at T0, T1, T2, T3. Thus, single bristle diameter is presumed to be a major determinant of the bristle stiffness of a PBT toothbrush. 

Nylon toothbrushes may be more effective in plaque removal than natural toothbrushes, because nylon bristles have higher stiffness than natural bristles [18]. However, nylon bristles absorb water in wet conditions, which reduces the bristle stiffness [19]. In contrast, PBT is a thermoplastic polyester compound that does not absorb water as much as nylon bristles [20]. Therefore, PBT is a useful plastic material for wet applications such as in toothbrushes. Additionally, PBT is resistant to heat and chemicals [20]. Thus, PBT bristles may be a good substitute for nylon bristles. However, it remains unknown whether PBT toothbrushes can maintain a consistent bristle stiffness for longer than nylon toothbrushes.

Several previous clinical studies have investigated the relationship between bristle splaying and plaque removal efficacy after toothbrush use [2,3,4,21,22,23,24,25]. In our earlier study, we found a significant reduction in the plaque removal efficacy of PBT toothbrushes after two months of use [4]. It is likely that bristle splaying of PBT toothbrushes is associated with plaque removal efficacy. Furthermore, physical properties other than bristle splaying (e.g., bristle stiffness) may have a significant impact on plaque removal efficacy. In this study, we investigated the change in bristle stiffness after several months of use. Despite the difference in the monofilament diameter in medium and soft toothbrushes, we found that a significant reduction in the bristle stiffness of the two types of toothbrushes occurred at almost the same time. This finding suggests that the difference in monofilament diameter may not have had an influence on the timing of the reduction of bristle stiffness in medium and soft toothbrushes. Bristle stiffness appears to be maintained in soft and medium PBT toothbrushes for two months. In contrast, a significant increase in bristle splaying was observed in the two types of toothbrushes after one month of use. Therefore, a significant reduction in bristle stiffness is presumed to occur almost one month after the bristles are significantly splayed. Considering that the plaque removal efficacy of soft and medium PBT toothbrushes was reduced after two months of use, PBT manual toothbrushes may become less effective for plaque removal at nearly the same time as the reduction in bristle stiffness [4]. The bristle stiffness of PBT toothbrushes may be importantly associated with plaque removal efficacy. 

The American Dental Association recommends that toothbrushes are replaced approximately every 3–4 months [26]. Long-term toothbrush use for six months reduces the efficacy of plaque removal [27]. Thus, it is commonly recommended that toothbrushes should be replaced at three-month intervals [28]. The use of worn toothbrushes with reduced bristle stiffness may cause gingival inflammation due to the retention of plaque adjacent to the gingival margin. Therefore, it is necessary to replace toothbrushes regularly to ensure effective plaque control. Based on the results of this study, it may be recommended that the PBT toothbrushes used in this study are replaced every two months because of reduced bristle stiffness and plaque removal efficacy. However, the relationship between the change in bristle stiffness and plaque removal efficacy remains unknown in other types of PBT toothbrushes from different manufacturers. Further study is required to clarify the relationship between bristle stiffness and plaque removal efficacy in manual PBT toothbrushes.

## 5. Conclusions

There was a significant reduction in the bristle stiffness of medium and soft PBT toothbrushes after two months of use. Considering the reduced bristle stiffness and plaque removal efficacy, it is recommended that the PBT toothbrushes used in this study are replaced at least every two months.

## Figures and Tables

**Table 1:** Changes in bristle stiffness of soft and medium toothbrushes.

Soft Type Toothbrush		Medium Type Toothbrush	
	Mean ± SD (N/cm^2^)	Mean ± SD (N/cm^2^)	*p* Value ^†^
T0	3.89 ± 0.29	4.52 ± 0.30	*p* < 0.001
T1	3.87 ± 0.30	4.50 ± 0.32	*p* < 0.001
T2	3.63 ± 0.29 **	4.33 ± 0.26 *	*p* < 0.001
T3	3.62 ± 0.32 **	4.18 ± 0.25 ***	*p* < 0.001

The bristle stiffness of soft and medium toothbrushes was significantly lower at T2 and T3 than at T0 (* *p* < 0.05, ** *p* < 0.01, *** *p* < 0.001, Tukey′s HSD test). There was a significant difference in the bristle stiffness between the groups at T0, T1, T2, and T3 (^†^ Student’s *t*-test).

**Table 2:** Changes in wear index of soft and medium toothbrushes.

Soft Type Toothbrush		Medium Type Toothbrush	
	Mean ± SD (mm)	Mean ± SD (mm)	*p* Value ^†^
T0	0.27 ± 0.11	0.13 ± 0.07	*p* < 0.001
T1	0.53 ± 0.26 ***	0.29 ± 0.18 ***	*p* < 0.001
T2	0.58 ± 0.32 ***	0.43 ± 0.19 ***	*p* < 0.05
T3	0.76 ± 0.39 ***	0.54 ± 0.39 ***	*p* < 0.01

The wear index of soft and medium toothbrushes was significantly greater at T1, T2, and T3 than at T0 (*** *p* < 0.001, Mann–Whitney U test with Bonferroni correction). There was a significant difference in the wear index of bristles between the groups at T0, T1, T2, and T3 (^†^ Mann–Whitney U test).
